# CD4 T-cell immune stimulation of HER2 + breast cancer cells alters response to trastuzumab in vitro

**DOI:** 10.1186/s12935-020-01625-w

**Published:** 2020-11-10

**Authors:** Patrick N. Song, Ameer Mansur, Kari J. Dugger, Tessa R. Davis, Grant Howard, Thomas E. Yankeelov, Anna G. Sorace

**Affiliations:** 1 Department of Radiology, The University of Alabama at Birmingham, 1670 University Blvd, Birmingham, AL 35233 USA; 2 Department of Biomedical Engineering, The University of Alabama at Birmingham, 1670 University Blvd, Birmingham, AL 35233 USA; 3 Department of Clinical and Diagnostic Sciences, The University of Alabama at Birmingham, Birmingham, AL USA; 4 Department of Biomedical Engineering, The University of Texas at Austin, Austin, TX USA; 5 Department of Chemical Engineering, The University of Texas at Austin, Austin, TX USA; 6 Department of Diagnostic Medicine, The University of Texas at Austin, Austin, TX USA; 7 Department of Oncology, The University of Texas at Austin, Austin, TX USA; 8 Livestrong Cancer Institutes, The University of Texas at Austin, Austin, TX USA; 9 Oden Institute for Computational Engineering and Sciences, The University of Texas at Austin, Austin, TX USA; 10 O’Neal Comprehensive Cancer Center, The University of Alabama at Birmingham, Birmingham, AL USA

**Keywords:** CD4 + T-cell, Co-culture, Herceptin, Live-cell imaging, TNF-α

## Abstract

**Introduction:**

The HER2 + tumor immune microenvironment is composed of macrophages, natural killer cells, and tumor infiltrating lymphocytes, which produce pro-inflammatory cytokines. Determining the effect of T-cells on HER2 + cancer cells during therapy could guide immunogenic therapies that trigger antibody-dependent cellular cytotoxicity. This study utilized longitudinal in vitro time-resolved microscopy to measure T-cell influence on trastuzumab in HER2 + breast cancer.

**Methods:**

Fluorescently-labeled breast cancer cells (BT474, SKBR3, MDA-MB-453, and MDA-MB-231) were co-cultured with CD4 + T-cells (Jurkat cell line) and longitudinally imaged to quantify cancer cell viability when treated with or without trastuzumab (10, 25, 50 and 100 μg/mL). The presence and timing of T-cell co-culturing was manipulated to determine immune stimulation of trastuzumab-treated HER2 + breast cancer. HER2 and TNF-α expression were evaluated with western blot and ELISA, respectively. Significance was calculated using a two-tailed parametric *t*-test.

**Results:**

The viability of HER2 + cancer cells significantly decreased when exposed to 25 μg/mL trastuzumab and T-cells, compared to cancer cells exposed to trastuzumab without T-cells (*p* = 0.01). The presence of T-cells significantly increased TNF-α expression in trastuzumab-treated cancer cells (*p* = 0.02). Conversely, cancer cells treated with TNF-α and trastuzumab had a similar decrease in viability as trastuzumab-treated cancer cells co-cultured with T-cells (*p* = 0.32).

**Conclusions:**

The presence of T-cells significantly increases the efficacy of targeted therapies and suggests trastuzumab may trigger immune mediated cytotoxicity. Increased TNF-α receptor expression suggest cytokines may interact with trastuzumab to create a state of enhanced response to therapy in HER2 + breast cancer, which has potential to reducing tumor burden.

## Introduction

Twenty-five percent of newly diagnosed breast cancer cases will overexpress the human epidermal growth factor receptor 2 (HER2) gene [1]. Preclinical and clinical studies indicate that the immune microenvironment of HER2 + tumors is driven by tumor infiltrating lymphocytes and macrophages, which can produce pro-inflammatory cytokines [2–4]. Immune inflammatory cytokine expression has been demonstrated to influence tumor progression and proliferation, highlighting the interplay between immune interaction, tumor phenotype, and progression [2, 3].

Trastuzumab is a clinically approved humanized monoclonal antibody that prevents the dimerization of the HER2 receptor [5, 6]. Trastuzumab is a key component in the treatment of primary and metastatic HER2 + breast cancer, improving progression-free survival compared to chemotherapy alone [7–10]. Trastuzumab has been observed to exhibit immunogenic qualities through increased granzyme release and natural killer cell activation [11–17]. Lee et al*.* found patients with increased tumor infiltrating leukocytes (TIL) responded more favorably to trastuzumab, suggesting that TILs may serve as a biomarker to identify which HER2 + breast cancer patients would most benefit from trastuzumab [16]. Moreover, Gagliato et al. found that patients with increased TIL were associated with decreased tumor recurrence [14]. Preclinically, trastuzumab has been observed to increase CD11c and F4/80 (markers of dendritic cells and macrophages, respectively) in in vivo models of HER2 + breast cancer, highlighting the immunogenic potential of anti-HER2 therapy [15]. Moreover, FcγR-mediated stimulation of CD4 + T-cells and activation of CD4 + T-cells with HER2-primed dendritic cell vaccines reduced tumor burden through tumor-specific T-cell response [18, 19]. Although clinical studies have shown that successful trastuzumab therapy is associated with immune cell infiltration, there exists a lack of longitudinal studies that examine trastuzumab-induced CD4 + immune interaction with HER2 + breast cancer [20].

Culturing of cancer cells with immune cells, or onco-immune co-culturing, has been used to study immune interactions between cancer cells and tumor associated macrophages (TAMs) [21–23]. Castellaro et al. co-cultured MCF-7 breast cancer cells with TAMs and found TAMs promoted cell proliferation and metastasis. Furthermore, Castellaro et al*.* found macrophages increased breast cancer’s resistance to tamoxifen, highlighting the interaction between immune cell presence and response to therapy [21]. While onco-immune co-culturing has been studied with cancer cells and TAMs, to our knowledge, the impact of T-cells on HER2 + breast cancer and subsequent longitudinal response to anti-HER2 therapy has not yet been investigated.

The purpose of this study is to investigate T-cell influence on HER2 + breast cancer in response to anti-HER2 trastuzumab therapy. We hypothesize that time resolved microscopy of CD4 + T-cell influence on trastuzumab treated HER2 + breast cancer will highlight the interaction between immune cells and cancer cell response to therapy. This study used longitudinal live cell imaging to quantify the effect of immune cell presence on trastuzumab-treated HER2 + breast cancer through in vitro co-culturing of CD4 + T-cells and HER2 + cancer cell lines (as seen in Fig. 4). This data has potential to serve as the foundation for guiding future studies in immune-based modulation to enhance response to targeted therapies in vivo.

## Methods

### Cell culture

HER2 + breast cancer cell lines (BT474, SKBR3, MDA-MB-453), HER2- breast cancer cell line (MDA-MB-231), and CD4 + T-cell line (Jurkat) were obtained from ATCC (Manassas, VA, USA). BT474 cells were grown in improved minimal essential media (Invitrogen, Carlsbad, CA) with 10% FBS and 1% insulin. SKBR3 cells were grown in McCoy’s 5A media with 10% FBS and 2 mM L-glutamine. MDA-MB-453 cells were grown in Leibovitz’s L-15 media (Sigma, St. Louis, MO, USA) with 10% FBS. MDA-MB-231 cells were grown in Dulbecco’s minimal essential media (Gibco, Gaithersburg, MD, USA) with 10% FBS, 2 mM L-glutamine, and 1 mM sodium pyruvate. Jurkat T-cells were grown in RPMI-1640 media (Gibco, Gaithersburg, MD, USA) with 10% FBS and 2 mM L-glutamine. Co-culturing of cancer cells and T-cells were conducted by suspending cancer cells and T-cells in cancer cell’s respective growth medium prior to plating.

### Fluorescence transfection of breast cancer cell lines

The SKBR3 cell line was transfected to express green fluorescent protein (GFP). A GFP plasmid was cloned into a Sleeping Beauty compatible vector (Addgene plasmid #60525). The GFP plasmid was co-transfected with pCMV (CAT) T7-SB100 Sleeping Beauty transposase (Addgene plasmid #34879) with Lipofectamine LTX (Thermo Fisher Scientific, Waltham, MA, USA). SKBR3 cells were selectively cultured with McCoy’s 5A media supplemented with 10% FBS, 1% L-glutamine and 200 µg/mL geneticin. Fluorescence activated cell sorting was used to separate cells and the highest 25% of GFP expressing cells were used in experiments. The pCMV (CAT)T7-SB100 plasmid and the pSBbie-Neo were gifts from Zsuzsanna Izsvak and Eric Kowarz, respectively [24, 25]. Fluorescence ubiquitination cell cycle indicator (FUCCI) transfected MDA-MB-453 and MDA-MB-231 cell lines were a generous gift from Dr. Vito Quaranta.

### Live cell imaging and image analysis

Viable cells were engineered to express fluorescence and cancer cell viability was determined by quantifying change in fluorescence signal (see Additional file 1: Figure S1). In vitro experiments examining treatment response in HER2 + breast cancer cell lines, T-cell influence on HER2 + breast cancer response to trastuzumab, and evaluation of timing of T-cell introduction on trastuzumab treated HER2 + breast cancer were carried out for approximately 144 h on 96-well glass bottom plates (Fisher Scientific. Catalog #165305) with an IncuCyte S3 imaging system (Essen Bioscience, Sartorius, Germany). Preliminary experiments were conducted to determine seeding density of cancer cells to facilitate longitudinal cell growth (BT474: 20,000 cells/well. SKBR3: 7500 cells/well. MDA-MB-453: 25,000 cells/well. MDA-MB-231: 1000 cells/well. Jurkat T-cells: 7000 cells/well). Cell seeding densities that resulted in continuous exponential growth and ~ 80% confluence at the final imaging timepoint were used. To retain CD4 + T-cells in co-cultured wells during treatment, plates were centrifuged at 500 *g* for 5 min prior to treatment and drug removal. For imaging, phase contrast and fluorescence images were collected every 3–6 h using 10 × magnification (Excitation/emission: 440–480/504–544 nm for green channels and 565–605/625–705 nm for red channels). Phase confluence and fluorescence data was analyzed using the IncuCyte S3 Live-Cell Image Analysis System. Cells were counted by automated image analysis using background subtraction and brightness threshold (2 green calibrated units and 0.8 red calibrated units). Mean values were summarized by averaging replicates at specified timepoints and percent change was determined by ((*X*_*1*_-*X*_*0*_)/*X*_*0*_) 100, where *X*_*0*_ and *X*_*1*_ represent cell viability at baseline and cell viability at subsequent timepoints, respectively.

#### Evaluation of treatment response to HER2 + breast cancer cell lines in vitro

**Fig.1:**
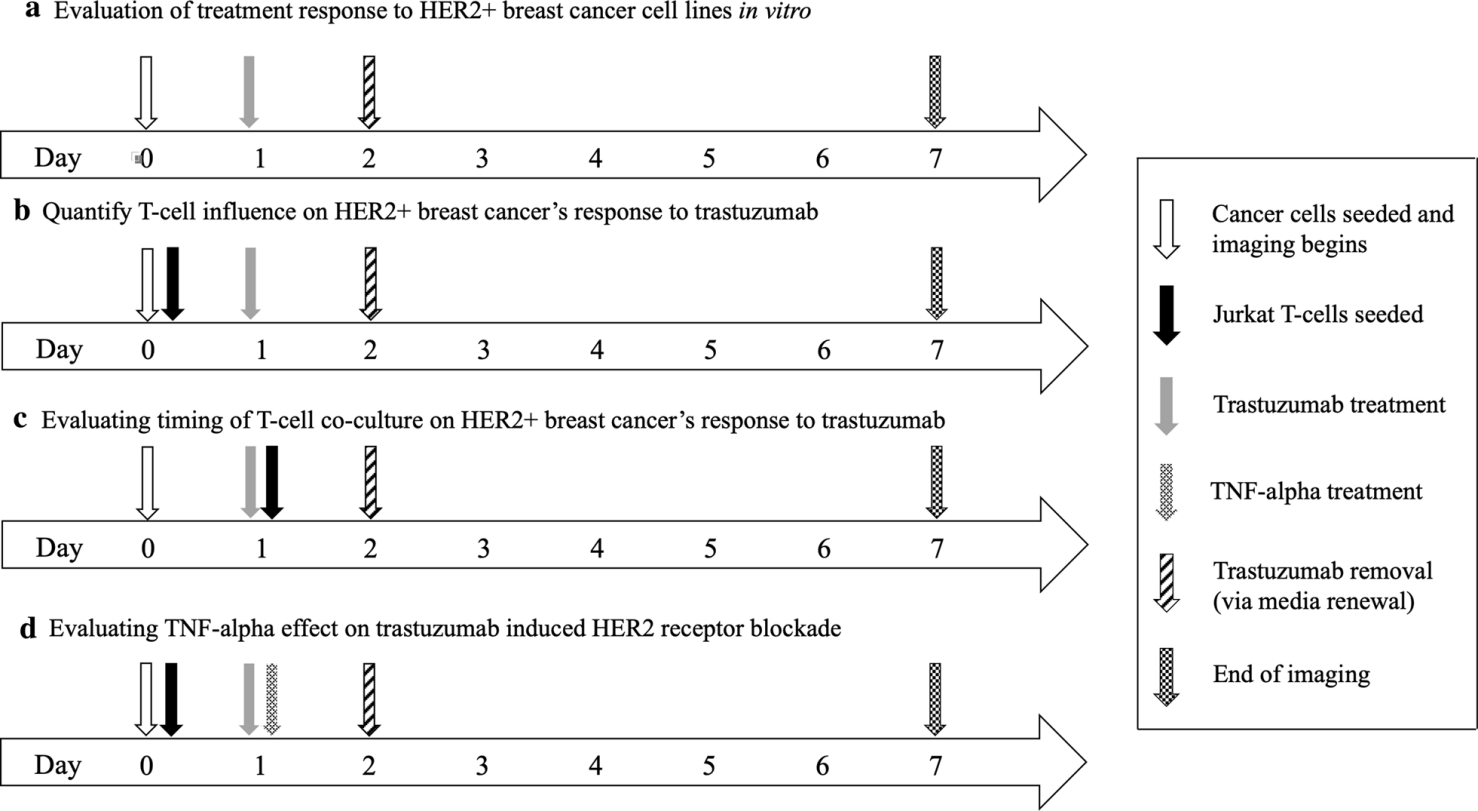
Timeline of in vitro experiments. **a** Treatment response of HER2 + breast cancer cells in response to incremental doses of trastuzumab (0–100 µg/mL) for 24 h. **b** Quantifying immune influence of CD4 + T-cells on HER2 + breast cancer in response to single agent trastuzumab. **c** Evaluating timing of T-cell co-culture on HER2 + breast cancer’s response to trastuzumab. CD4 + T-cells were introduced into cell culture at *t* = 0 h during initial plating of cells or *t* = 24 h during trastuzumab treatment and longitudinal changes in cell viability was assessed. **d** Evaluating TNF-α effect on trastuzumab induced HER2 receptor blockade

**Fig.2:**
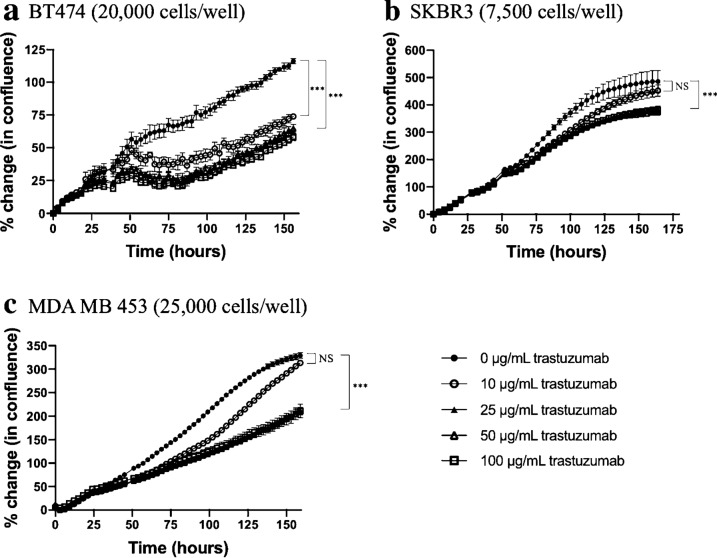
In vitro treatment response of HER2 + breast cancer to single agent trastuzumab. HER2 + breast cancer cells were treated with incremental doses of single agent trastuzumab therapy and longitudinal changes in cell confluence was observed over 7 days. In comparison to control groups, cancer cells treated with 25, 50 and 100 µg/mL were statistically similar (*p* = 0.38, 0.72 and 0.89 in BT474, SKBR3 and MDA-MB-453 cell lines, respectively) to one another and 25 µg/mL was used for subsequent experiments

To quantify HER2 + breast cancer response to trastuzumab, cell lines were treated with trastuzumab and viability was assessed (Fig. 1a). GFP BT474, GFP SKBR3, and FUCCI MDA-MB-453 cells were plated in 96-well plates. On day 1 (24 h after cell seeding), cells were treated with trastuzumab (10, 25, 50 and 100 µg/mL). On day 2, trastuzumab was removed through media change and cells were longitudinally observed for five additional days (see Fig. 2). Percent change in cancer cell viability was normalized to initial confluence. Mean confluence was summarized by averaging confluence at specified timepoints and percent change was determined. Each treatment group has 4–8 replicates.

#### T-cell influence on HER2 + breast cancer’s response to trastuzumab

To test whether T-cell co-culture affects cancer cell response to trastuzumab, HER2 + (BT474, SKBR3 and MDA-MB-453) and HER2- (MDA-MB-231) cells were co-cultured with T-cells on a 96 well plate. On day 1, cells were treated with 25 µg/mL trastuzumab. On day 2, trastuzumab was removed through media renewal and replaced with fresh media (without trastuzumab). Following trastuzumab treatment, cells were longitudinally observed for five additional days (Fig. 1b). Changes in cancer cell viability of co-cultured cells was normalized to that of initial cell viability on day 0 and the fold change per replicate was correlated to HER2 western blot. Each treatment group has 4–8 replicates.

#### Evaluating timing of T-cell co-culture on HER2 + breast cancer’s response to trastuzumab

To determine whether timing of immune stimulation of HER2 + breast cancer cells in response to trastuzumab impacts longitudinal treatment response, the timing of when T-cells were introduced to BT474 cell culture was examined (Fig. 1c). T-cells were co-cultured with BT474 cancer cells either during initial cell seeding on day 0 or day 1. On day 1, groups were treated with trastuzumab (25 µg/mL) and T-cells through media change. On day 2, trastuzumab was removed through media renewal. Following trastuzumab treatment, cells were longitudinally observed for five additional days. Changes in cell viability was normalized to initial cell viability. Each group has 3 replicates.

#### Evaluating TNF-α effect on trastuzumab induced HER2 receptor blockade

Experiments evaluating the effects of tumor necrosis factor-alpha (TNF-α) on trastuzumab-induced HER2 receptor blockade were carried out for approximately 144 h with an EVOS M7000 imaging system (ThermoFisher, Waltham, MA, USA). Phase contrast and fluorescence images were collected every 8 h using 4 × magnification (Excitation/emission: 470/525 nm for green channels). Images were analyzed with MATLAB image analysis code to quantify the number of fluorescent objects per field of view.

To determine whether TNF-α cytokine expression impacts cancer cell viability during trastuzumab induced HER2 receptor blockade, cancer cells with high overexpression of HER2 were treated with trastuzumab and TNF-α and cellular response was longitudinally monitored (Fig. 1d). BT474 and SKBR3 cells were co-cultured with T-cells on a 96 well plate. On day 1, cells were treated with either (1) control, (2) 25 µg/mL trastuzumab, (3) 100 ng/mL human recombinant TNF-α (R&D Systems. Catalog #: 210-TA-005), (4) 25 µg/mL trastuzumab + 100 ng/mL TNF-α, (5) T-cells or (6) T-cells + 25 µg/mL trastuzumab. On day 2, treatment was removed through media renewal. Following trastuzumab treatment, cells were longitudinally observed for changes in viability for five additional days. Changes in the number of fluorescent objects were used to determine changes in cancer cell viability. Each treatment group has 4–5 replicates.

### HER2 and TNF-α quantification

BT474, SKBR3, MDA-MB-453, and MDA-MB-231 cancer cells were washed with cold PBS and lysed. Lysates were centrifuged and collected for quantification with a Nanodrop 2000c spectrophotometer (Thermo Fisher Scientific, Waltham, MA, USA). 20 µg of protein per cell line was run on a 4–12% NuPAGE Bis–Tris gel (Invitrogen, Carlsbad, CA) and transferred to a PVDF membrane. The membrane was blocked, probed with HRP conjugated mouse anti-human β-actin overnight at 4° C and developed with Amersham ECL western blot detection system (GE healthcare, Buckinghamshire, UK). Membranes were developed and visualized with an SRX-101A Medical Film Processor (Konica Minolta Medical and Graphic, Inc., Shanghai, China). After β-actin was used as a control to confirm consistent protein levels, the membrane was stripped and probed with 1:1000 rabbit anti-human HER2/ErbB2 primary antibody (Cell Signaling Technology, Danvers, MA, USA. Catalog no. #2242) and 1:1000 rabbit anti-human TNF-α primary antibody (Cell Signaling Technology, Danvers, MA, USA. Catalog no. #C25C1) overnight at room temperature. The membrane was washed, incubated with 1:2000 HRP conjugated goat anti-rabbit IgG secondary antibody (Cell Signaling Technology. Catalog no. #7074) for 1 h at room temperature. The membrane was redeveloped and visualized for protein expression. Bands were analyzed with the Image Studio Lite (LI-COR Biosciences, Lincoln, NE, USA). Individual cell line HER2 and TNF-α expression was normalized to β-actin expression.

### TNF-α expression

BT474, T-cells, and a co-culture of BT474 and T-cells were longitudinally imaged (N = 3 wells per group) to correlate changes in cancer cell viability with TNF-α expression. All experimental groups were either treated with 25 µg/mL trastuzumab or media (control) at *t* = 24 h. Treatment was removed at *t* = 48 h. On days 0 and 7, supernatant was collected for ELISA analysis. A TNF-α ELISA kit (LSBio. Catalog No. LS-F2557-1) was used to quantify TNF-α expression per sample per well. ELISA expression was quantified with a Cytation5 microscope (BioTek Instruments, Winooski, VT). Samples were normalized to expression on day 0. Samples were processed individually and averaged per timepoint. Because decreased cell viability was only observed in HER2 overexpressing cell lines, ELISA against TNF-α was only performed on BT474 cells due to their innate overexpression of HER2 protein.

### Statistical analysis

Cell viability in co-cultured groups was analyzed through longitudinal quantification of cell fluorescence (see Additional file 1: Figure S1). Groups were summarized by average confluence, average cell number ± standard error of mean (SEM). A Student’s t-test was used to assess group differences. Correlation of confluence and fluorescence was analyzed by computing the Pearson correlation coefficient. The Grubbs outlier test was used to eliminate any data points that were statistical outliers. A *p* value < 0.05 was considered statistically significant. All statistical analysis were completed with GraphPad Prism 7 (La Jolla, CA, USA).

## Results

### Longitudinal in vitro imaging reveals trastuzumab effects are saturated at higher concentrations

Figure 2 displays BT474, SKBR3, and MDA-MB-453 changes in cell viability in response to increasing doses of trastuzumab. BT474, SKBR3, and MDA-MB-453 control groups were observed to have 105.8 ± 20.9%, 446.5 ± 29% and 329 ± 19% increase in cell growth in 7 days, respectively. In groups treated with 10 µg/mL of trastuzumab, a significant decrease in cell growth compared to control on day 7 was only observed in BT474 cancer cells (*p* < 0.01). In groups treated with 10 µg/mL of trastuzumab, BT474 cancer cells were observed to have a 74.0 ± 3.6% increase in cell growth on day 7 when normalized to baseline cell growth on day 0 (*p* < 0.01), SKBR3 cancer cells were observed to have a 452.5 ± 60.6% increase in cell growth on day 7 (*p* = 0.48), and MDA-MB-453 cancer cells were observed to have 313.4 ± 14% increase in cell growth on day 7 (*p* = 0.06). In groups treated with 25 µg/mL trastuzumab, BT474 cancer cells were observed to have 61.9 ± 4.8% increase in cell growth on day 7 (*p* < 0.01), SKBR3 cancer cells were observed to have 384.8 ± 29.9% increase in cell growth on day 7 (*p* = 0.03), and MDA-MB-453 cancer cells were observed to have 213.3 ± 16% increase in cell growth on day 7 (*p* < 0.01).

BT474 cells treated with 25 µg/mL trastuzumab were observed to have 61.9 ± 4.8% increase in cell growth on day 7, statistically similar to the group treated with 100 µg/mL trastuzumab (*p* = 0.38). SKBR3 cells treated with 25 µg/mL trastuzumab were observed to have 384.8 ± 29.9% cell growth on day 7, statistically similar to the group treated with 100 µg/mL trastuzumab (*p* = 0.72). MDA-MB-453 cancer cells treated with 25 µg/mL trastuzumab were observed to have 213.3 ± 16% cell growth on day 7, which is similar to the group treated with 100 µg/mL trastuzumab (*p* = 0.89). All trastuzumab doses above 25 µg/mL were statistically similar (*p* > 0.05) showing no additional cytotoxic benefit, therefore 25 µg/mL was used for all future experiments.

### T-cell co-culture increases trastuzumab efficacy in HER2 + breast cancer with respect to HER2 expression

**Fig.3:**
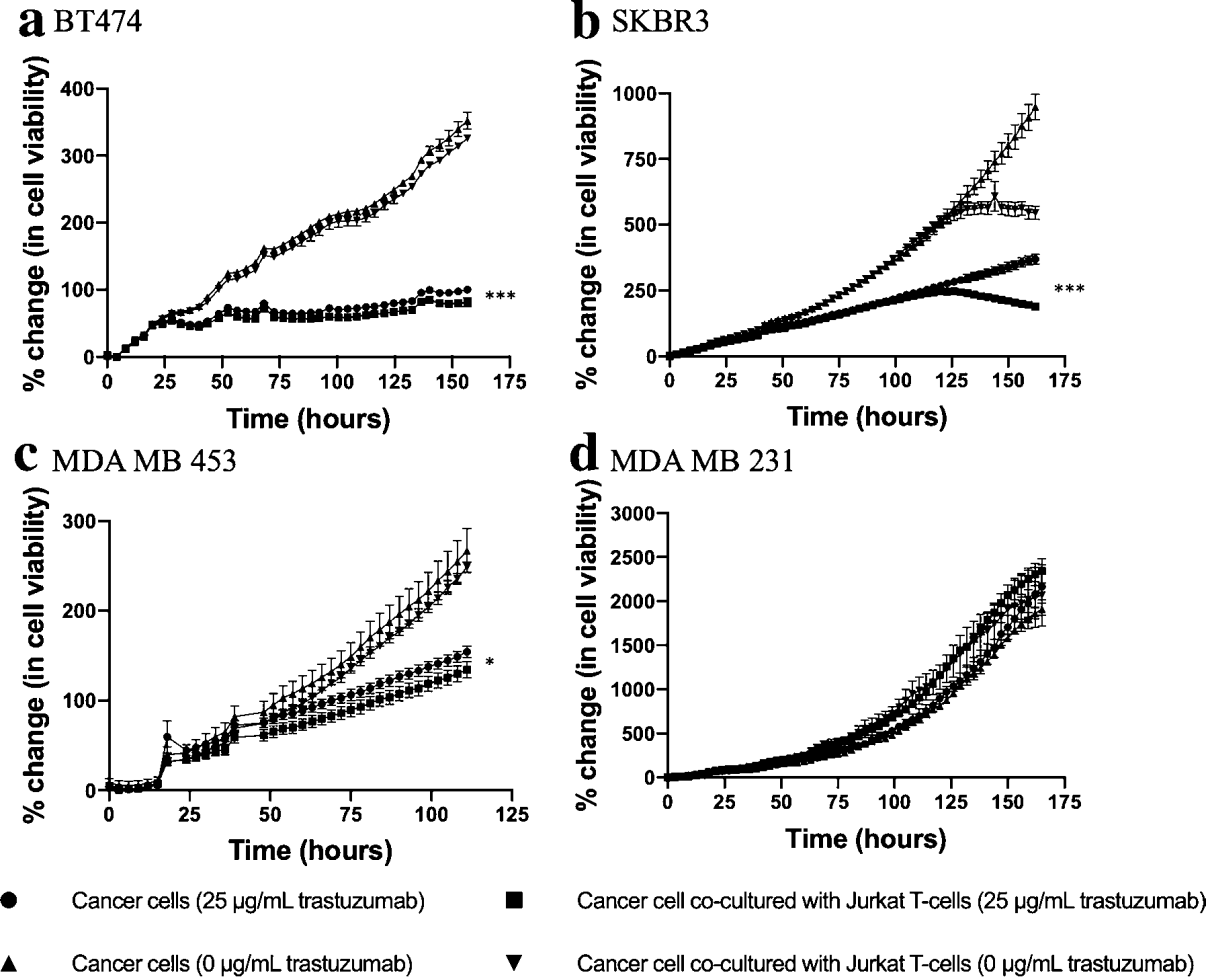
Immune stimulation of trastuzumab treated HER2 + breast cancer cells. Normalized percent change in confluence of **a** BT474, **b** SKBR3 and **c** MDA-MB-453 and **d** MDA-MB-231 breast cancer cells in response to 25 µg/mL trastuzumab and CD4 + T-cell presence. Significant differences in cell viability between (1) trastuzumab treated cancer cells and (2) trastuzumab treated co-cultured cells was only observed in BT474 and SKBR3 cells (*p* = 0.01). Decreased cell viability between (1) trastuzumab treated cancer cells and (2) trastuzumab treated co-cultured cells was only observed in MDA-MB-453 cells was observed but was only trending toward significance (*p* = 0.08). Error bars are representative of standard error of mean (SEM)

**Fig.4:**
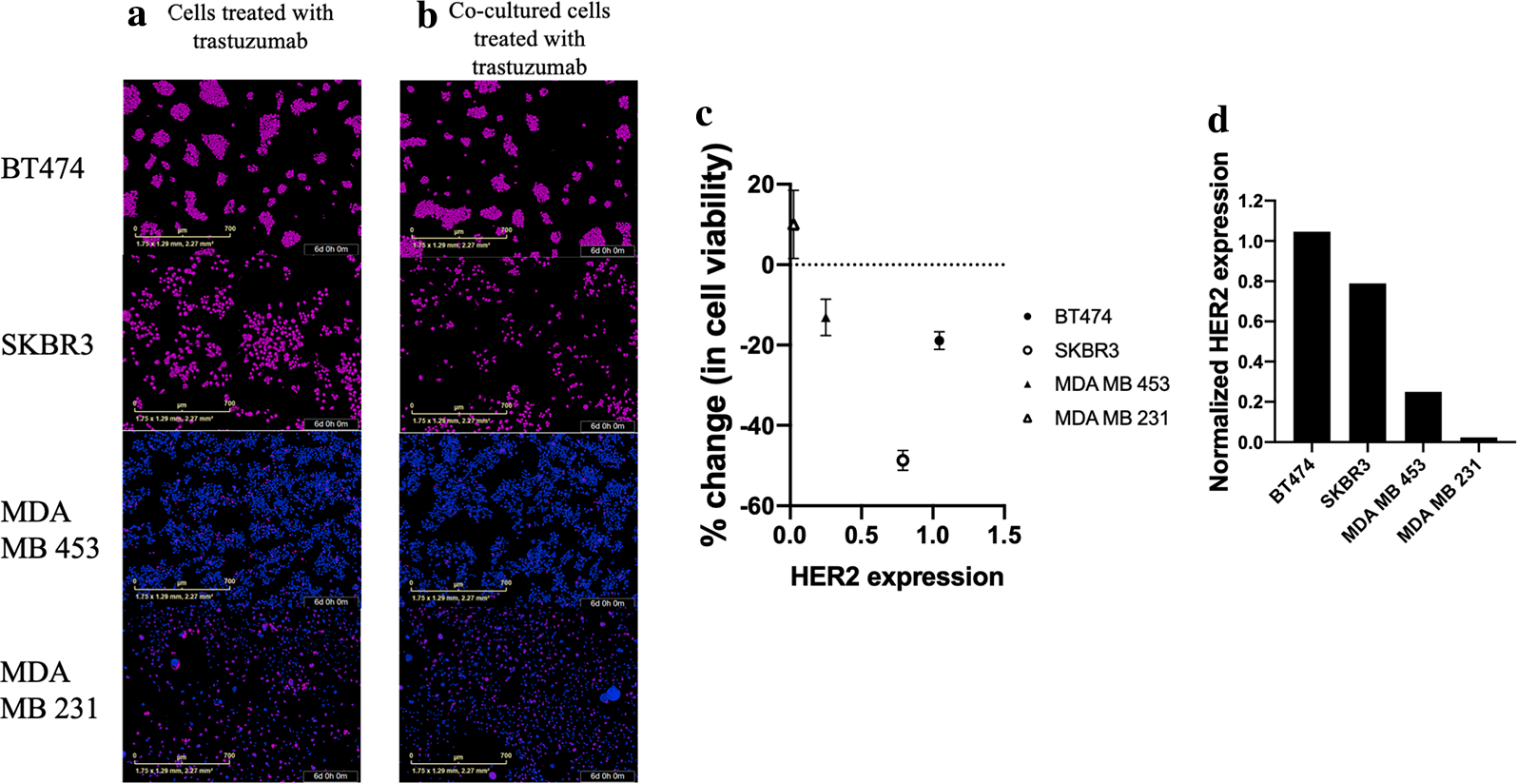
Representative images of T-cell and cancer cell co-culturing with fluorescence segmentation and HER2 quantification. GFP and RFP segmented images of breast cancer cells treated with 25 µg/mL trastuzumab when cultured without (**a**) and with (**b**) CD4 + T-cells. **c** Correlation of HER2 expression and significance between changes in viability of trastuzumab treated cancer cells and trastuzumab treated co-cultured cells. The fold change in cell viability has a negative correlation to HER2 expression (*r*^2^ = 0.49). **d** Normalized HER2 expression was identified with western blots. High HER2 overexpression was observed in BT474 and SKBR3 cell lines. Moderate HER2 overexpression was observed in MDA-MB-453 cell lines. Low HER2 overexpression was observed in MDA-MB-231 cell lines

Figure 3 displays BT474, SKBR3, MDA-MB-453 and MDA-MB-231 cell viability in response to trastuzumab (25 µg/mL) in the presence or absence of T-cells. When treated with trastuzumab, BT474 cancer cells demonstrated 100.2 ± 3% increase in cell viability on day 7, while BT474 cancer cells co-cultured with T-cells demonstrated significantly less cell viability, revealing an 81.4 ± 6.9% increase in cell viability on day 7 (*p* = 0.01). When treated with trastuzumab, SKBR3 cancer cells demonstrated 368 ± 49% increase in cell viability on day 7, while SKBR3 cancer cells co-cultured with T-cells demonstrated significantly less cell viability, revealing a 178.6 ± 39% increase in cell viability on day 7 (*p* = 0.01, Fig. 3). When treated with trastuzumab, MDA-MB-453 cancer cells demonstrated 154.4 ± 15.7% increase in cell viability on day 7, while MDA-MB-453 cells co-cultured with T-cells demonstrated decreased cell viability trending toward significant, revealing a 134.4 ± 21% increase in cell viability on day 7 (*p* = 0.09). When treated with trastuzumab, MDA-MB-231 cancer cell demonstrated 2162 ± 340% increase in cell viability on day 7, while MDA-MB-231 cancer cells co-cultured with T-cells demonstrated 2342.6 ± 312% increase in cell viability on day 7 (*p* = 0.40). Additional file 2: Figure S2 displays HER2 receptor expression with western blot in breast cancer cell lines used in this experiment. Figure 4 shows how BT474, SKBR3, MDA-MB-453 and MDA-MB-231 breast cancer cells exhibited 0.49, 0.41, 0.24 and 0.01 fold HER2 expression, respectively, when normalized to β-actin. Decreases in endpoint cancer cell viability in the presence of T-cells and trastuzumab correlates with HER2 receptor expression (*r*^2^ = 0.59, Fig. 4c).

**Fig.5:**
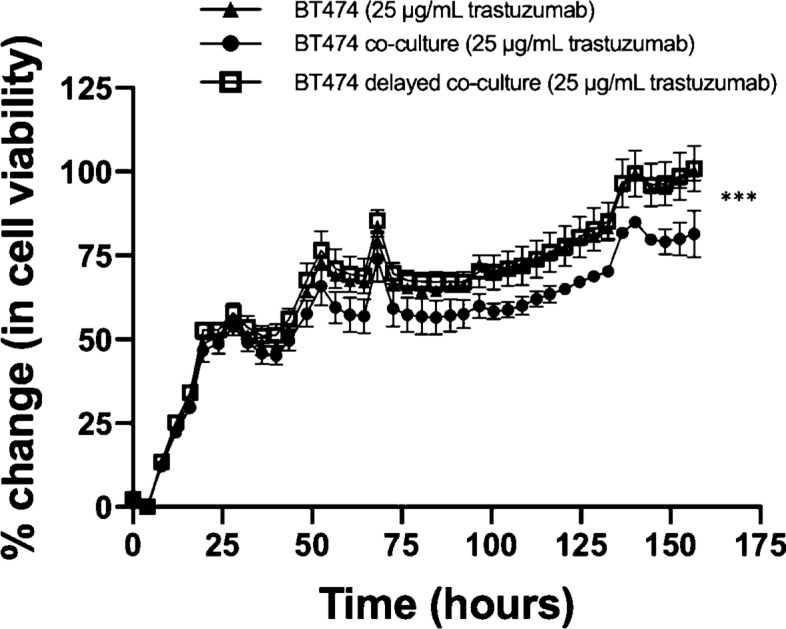
Longitudinal analysis of incubation time on efficacy of CD4 + T-cells to increase efficacy of targeted HER2 antibodies in BT474 cancer cells. To study the impact of T-cells on trastuzumab response in HER2 + breast cancer, T-cells were introduced to cell culture at two different times: *t* = 0 and *t* = 24 h. Significant differences in cell viability were only observed when T-cells were introduced into cell culture at *t* = 0 h (*p* = 0.01)

Figure 5 displays BT474 cell viability in response to trastuzumab (25 µg/mL) with T-cells co-cultured on day 0 (BT474 co-culture) or on day 1 (BT474 delayed co-culture). When BT474 cells were treated with trastuzumab on day 1, BT474 cells demonstrated a 100.2% ± 3% increase in cell viability on day 7. When T-cells were introduced to BT474 simultaneously with trastuzumab treatment on day 1, BT474 cells demonstrated a 100.9 ± 6.8% increase in cell viability on day 7, which was statistically similar to BT474 cells treated with trastuzumab (*p* = 0.88). When BT474 cells were cultured with T-cells on day 0 and treated with trastuzumab on day 1, the cells demonstrated an 81.4 ± 7% increase in cell viability on day 7, a significant decrease in viability compared to BT474 cells treated with trastuzumab (*p* = 0.01).

### TNF-α ELISA assay reveals increased expression in T-cells treated with trastuzumab

**Fig.6:**
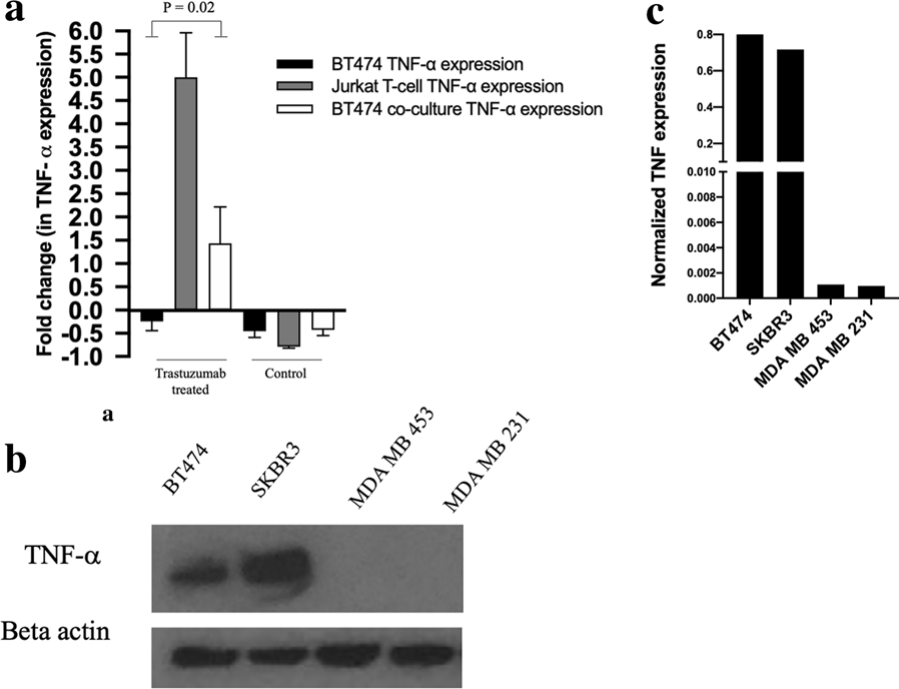
ELISA analysis of TNF-α expression in trastuzumab treated HER2 + breast cancer. **a** Fold change of TNF-α ELISA analysis from day 0 to 7 reveals an increase in TNF-α expression in trastuzumab treated cells cultured with CD4 + T-cells (*p* = 0.02). **b** Western blot of TNF-α and β-actin expression in BT474, SKBR3, MDA-MB-453 and MDA-MB-231 cell lines. **c** High TNF-α expression was observed in the BT474 cell line. Moderate TNF-α expression was observed in the SKBR3 cell line. Low TNF-α expression was observed in MDA-MB-453 and MDA-MB-231 cell lines. β-actin was also assayed and used to normalize protein expression

Figure 6a displays normalized TNF-α expression in (1) BT474 cancer cells, (2) T-cells, and (3) a co-culture of BT474 cancer cells and T-cells treated with trastuzumab. On day 7, BT474 cells alone, T-cells alone and co-cultured cells treated with trastuzumab had a 0 ± 20, 499.8 ± 96, 143.7 ± 78% increase in TNF-α expression, respectively. On day 7, control BT474 cells, control T-cells and control co-cultured cells exhibited a − 45 ± 13, − 78 ± 3, − 42 ± 12% increase in TNF-α expression, respectively. Trastuzumab treated CD4 + T-cells were observed to increase in TNF-α expression compared to control CD4 + T-cells (*p *< 0.01). Trastuzumab treated cancer cells co-cultured with T-cells had increased TNF-α expression compared to trastuzumab treated cancer cells without T-cells (*p* = 0.02). Figure 6b, c displays TNF-α receptor expression in HER2 + cell lines used in this study. BT474, SKBR3, MDA-MB-453 and MDA-MB-231 breast cancer cells exhibited 1.12, 0.72, 0.01 and 0.01 fold TNF-α fold expression when normalized to β-actin expression.

### TNF-α alters treatment response when treated in combination with trastuzumab

**Fig.7:**
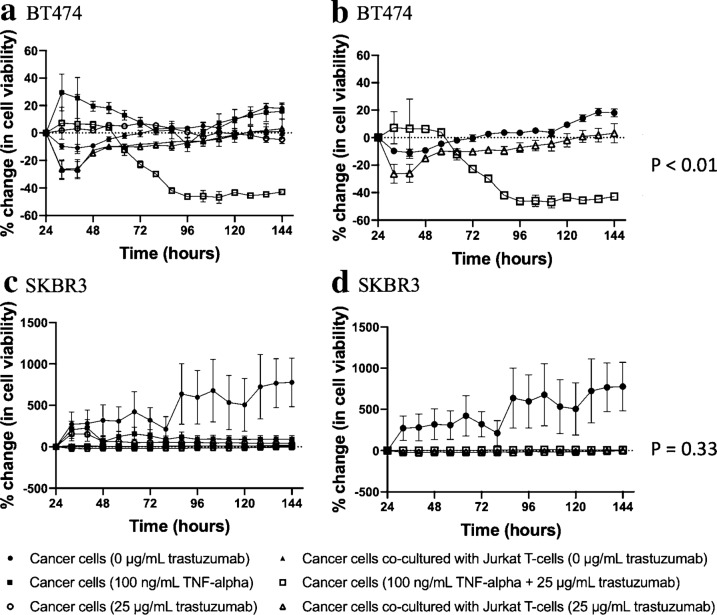
Longitudinal observation of TNF-α and trastuzumab on HER2 + breast cancer. Human recombinant TNF-α and trastuzumab was used to determine if decreased cell viability in co-cultured groups is the result of TNF-α receptor activation in conjunction with trastuzumab induced HER2 receptor blockade in **a** BT474 and **c** SKBR3 cell lines. Two HER2 + cell lines were used and both cell lines had similar responses in cancer cells treated with human recombinant TNF-α and trastuzumab vs trastuzumab treated cancer cells co-cultured with T-cells in **b** BT474 and **d** SKBR3 cell lines (*p* = 0.01 and 0.33, respectively)

Figure 7 displays changes in cell viability in response to trastuzumab and human recombinant TNF-α and notes that lowered cell viability in co-cultured groups treated with trastuzumab is related to TNF- α receptor expression. On day 7, control BT474 cells exhibited a 18 ± 6.6% increase in cell viability and TNF-α treated BT474 exhibited a 15.9 ± 13.4% increase in cell viability (*p *= 0.75). Trastuzumab treated BT474 cells co-cultured with T-cells exhibited a 3.2 ± 15.7% increase in cell viability and BT474 cells treated with trastuzumab and human recombinant TNF-α exhibited a − 42.9 ± 3.8% (*p* < 0.01). On day 7, control SKBR3 cells exhibited a 776.7 ± 589.1% increase in cell viability and TNF-α treated SKBR3 exhibited a 92.9 ± 97.3% (*p* = 0.03). Trastuzumab treated SKBR3 cells co-cultured with T-cells exhibited a − 1.7 ± 19.2% increase in cell viability and SKBR3 cells treated with trastuzumab and human recombinant TNF-α exhibited a 8.7 ± 3.3% increase in cell viability (*p* = 0.33).

## Discussion

This study seeks to investigate immune stimulation of HER2 + breast cancer in response to anti-HER2 trastuzumab therapy and identify potential mechanisms of enhanced response. It was observed that immune influence on HER2 + breast cancer in response to anti-HER2 targeted therapy increased treatment efficacy compared to cancer cells treated with trastuzumab without immune influence. Immune response to trastuzumab treated HER2 + breast cancer was shown to be impacted by CD4 + T-cell stimulation. Delaying the timing of T-cell co-culture by introducing T-cells 24 h after HER2 + breast cancer cells were seeded impacted the response of the cells to treatment. This significance was only observed in the cell lines highly overexpressing HER2, such as BT474 and SKBR3 (Fig. 4). Slight differences were observed in cancer cells treated with trastuzumab and co-cultured cells treated with trastuzumab in HER2 moderately overexpressing cell line, MDA-MB-453; however, this difference was only trending towards statistical significance, suggesting immune stimulation in trastuzumab treated HER2 + breast cancer cells is related to HER2 expression. The MDA-MB-231 cell line does not overexpress HER2 and lack of response to trastuzumab has been previously studied [26–29]. HER2 overexpression has been noted to increase tumor resistance to hormone-based therapy and certain chemotherapies [30, 31]; however, HER2 expression in relation to immune stimulation and trastuzumab response has not previously been characterized.

TNF-α serves a dual role in tumor proliferation and apoptosis. TNF-α has been shown to promote destruction of tumor vasculature and synergize with liposome-mediated chemotherapy [32–34]. Donato et al*.* used TNF-sensitive and TNF-resistant MCF-7 cell lines to study TNF-α treatment on poly (ADP-ribose) polymerase (PARP) cleavage and cell death [34]. Increased PARP cleavage and cell death was observed in TNF-sensitive MCF-7 cells, compared to TNF-resistant MCF-7 cells, suggesting TNF treatment increased DNA damage and apoptosis [34]. Conversely, TNF-α has also been observed to promote inflammation and tumor proliferation [35, 36]. Egberts et al. found that pancreatic cancer cell lines treated with TNF-α were observed to have increased invasive properties and invasion promoting proteins, such as interleukin-8 (IL-8) and matrix metallopeptidase-9 (MMP9) [35]. Similarly, increased expression of TNF-α was proportional to tumor grade and has also been observed in invasive breast ductal carcinomas. In our study, in vitro ELISA and longitudinal cell imaging suggest TNF-α expression improves efficacy of anti-HER2 therapy. Treatment of cancer cells with TNF-α and trastuzumab results in similar decreases in cell viability as trastuzumab-treated cancer cells co-cultured with T-cells in the high overexpressing HER2 + breast cancer cell lines, BT474 and SKBR3.

TNF-α expression has been studied in breast cancer response to therapy [37–39]. Lee et al*.* observed decreased cell viability in the MCF-7 cell line in response to TNF-α therapy, hypothesizing that TNF-α downregulates estrogen receptor expression [39]. While our study supports the role of TNF-α in decreasing cell viability, two of the HER2 + cell lines used in our study, SKBR3 and MDA-MB-453, do not overexpress estrogen receptors, suggesting cell viability changes may be multifaceted and not just the result of TNF-α induced estrogen receptor downregulation. Mercogliano et al*.* used plasmid transfection to generate TNF-α overexpressing HER2 + cell lines and found that TNF-α overexpression negatively correlated with trastuzumab resistance [38]. Differences in results from Mercogliano et al*.* and our study could be attributed to treatment incubation time and endpoint analysis. Mercogliano et al*.* measured changes in cell proliferation after 2 days post treatment, whereas our study measured longitudinal treatment response up to five days post treatment. Moreover, in our study, TNF-α induced changes in cell proliferation occurred approximately 3.5 days after treatment.

While T-cell immune stimulation has been studied in preclinical models of cancer, these studies have focused on changes in CD4 + T-cell viability in the presence of cancer cells [40, 41]. Zhu et al. used a MTT glucose metabolism assay to study CD4 + T-cell proliferation when co-cultured with SW480 colon cancer cells and observed that colorectal cancer cells increased apoptosis in lymphocytes [41]. Youssef et al. used annexin-V staining to determine the impact of cancer cells and oxygenation on T-cell viability and found increased CD4 + T-cell apoptosis when co-cultured with cancer cells in hypoxic conditions, implying cancer cell signaling interaction with immune cells [40]. To our knowledge, no other study has characterized the effect of immune stimulation and anti-HER2 targeted therapies on breast cancer cell viability.

Limitations of this study include the lack of comparative data with CD8 + T-cells. CD8 + cytotoxic T-cells are observed to target cancerous tissue and patients with high CD8 + T-cell infiltration have a significant increase in survival [42]. TALL-104, a CD8 + T-cell cell line, can be used as a future direction to study CD8 + T-cell influence on trastuzumab treated HER2 + breast cancer [43, 44]. An additional limitation of this study is the lack of in vivo data detailing changes in CD4 + T-cell immune infiltration. Future in vivo studies may provide additional data and could potentially be simulated through engrafting HER2 + breast cancer in a humanized mouse. Changes in immune infiltration can be determined through tracking of CD4 + T-cell localization with longitudinal noninvasive immuno-positron emission tomography (PET) imaging [45, 46]. Freise et al. previously reported a [^89^Zr] anti-CD4 cys-diabody to track CD4 + T-cell localization in vivo, with high uptake in the spleen, lymph nodes and thymus in normal non-tumor bearing mice [45]. Molecular mechanisms of enhanced response to trastuzumab in HER2 + breast cancer would be strengthened by additional cytokine analysis. Furthermore, multiple cytokine expression would be an interesting future direction to explore in an in vivo tumor model comparing CD4+ T-cell infiltration. Biological validation of T-cell immune infiltration can be achieved through flow cytometry against peripheral blood [20, 47–49]. Future experiments could investigate the involvement of the Fc-region of CD4 + T-cells and whether co-precipitation experiments can identify interaction between CD4 + T-cells and HER2 + breast cancer cells treated with trastuzumab. Similarly, future directions could include assessment of other cancer types (i.e. gastric cancer) that have high overexpression of HER2.

## Conclusion

This study uses in vitro longitudinal live cell imaging and fluorescent microscopy to track immune stimulation in trastuzumab treated HER2 + breast cancer through onco-immune co-culturing. Our data produced preliminary evidence that TNF-α activation could interplay with HER2 receptor activity to affect overall cell viability. Importantly, information from this work can be used to provide insight into immune interactions following trastuzumab therapy, used to identify mechanisms of enhanced response, and, in the future, to help guide combination therapies in vivo.

## Supplementary information

Additional file 1: **Figure S1.** Correlation of normalized change in in vitro fluorescent cells and confluence. Changes in number of fluorescent cells and changes in confluence were plotted using a Pearson Correlation Test (*r* = 0.98, *p* < 0.01). A linear relationship between change in fluorescent cells and changes in confluence was observed until approximately 250% increase in confluence

Additional file 2: **Figure S2.** Western blot of HER2 expression in breast cancer cell lines. Western blot of HER2 expression in breast cancer cell lines. Breast cancer cell lines were probed for HER2 expression through western blot. High HER2 expression was observed in BT474 and SKBR3 cell lines. Moderate HER2 expression was observed in MDA-MB-453 cell line. Low HER2 expression was observed in MDA-MB-231 cell line

## Data Availability

Datasets generated in this study are available from the corresponding author upon reasonable request.

## Abbreviations

HER: Human Epidermal Growth Factor Receptor
GFP: Green fluorescent protein
TNF-α: Tumor necrosis factor-alpha
TIL: Tumor infiltration leukocytes
ATCC: American Type Culture Collective
PBS: Phosphate buffer saline
ELISA: Enzyme Linked Immunosorbent Assay
MTT: 3-[4,5-Dimethylthiazole-2-yl]-2,5-diphenyltetrazolium bromide
MMP-9: Matrix metallopeptidase-9
PET: Positron emission tomography

## Code availability

Analysis code used in this study is available from the corresponding author upon request.
