# Supplementary figures and images for: CD4 T-cell immune stimulation of HER2 + breast cancer cells alters response to trastuzumab in vitro
Source: Cancer Cell Int. 2020 Nov 10;20:544. doi: 10.1186/s12935-020-01625-w (PMC7654187; doi:10.1186/s12935-020-01625-w)

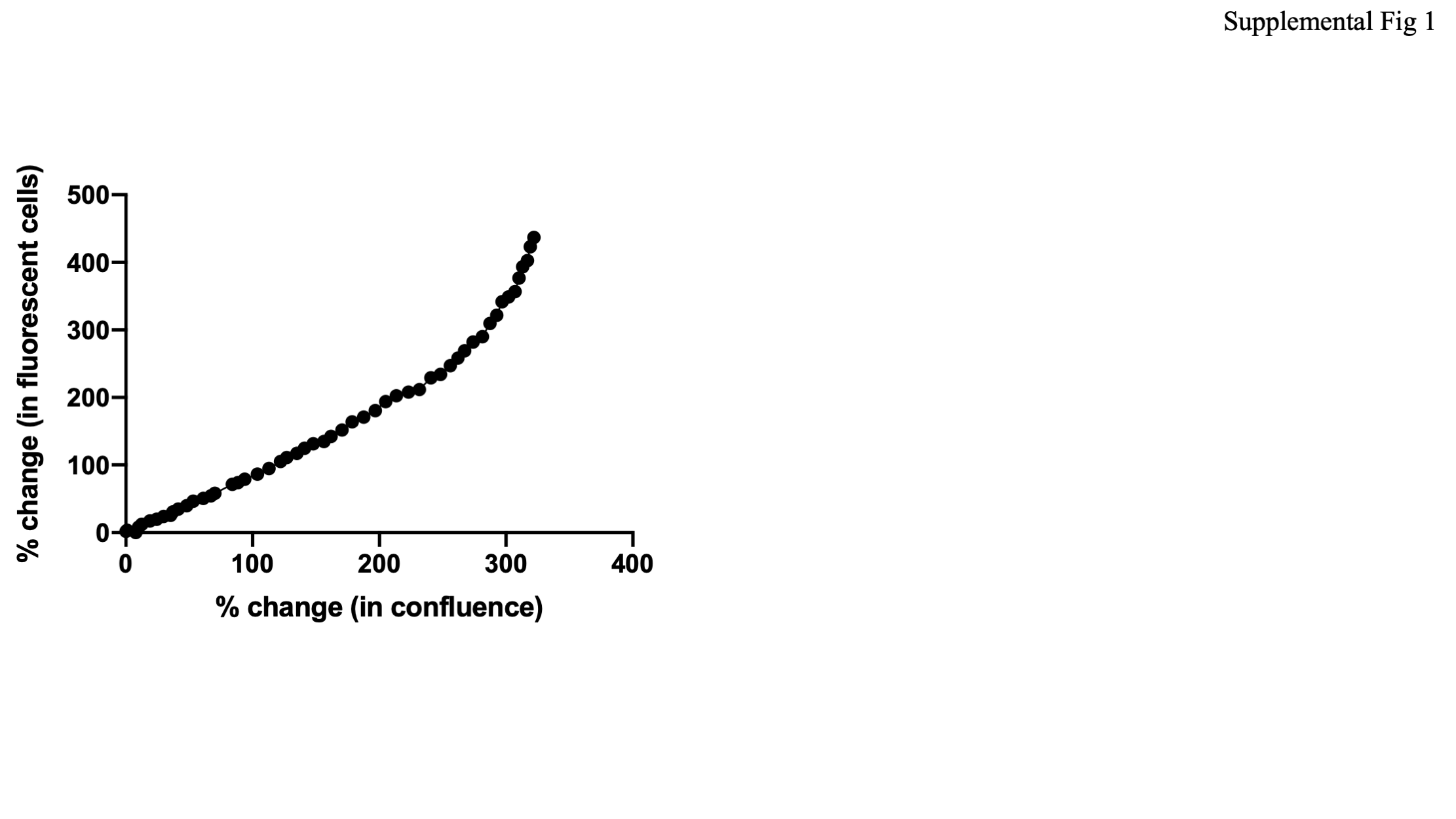

Supplement: Supplementary file 1 — Additional file 1: Figure S1. Correlation of normalized change in in vitro fluorescent cells and confluence. Changes in number of fluorescent cells and changes in confluence were plotted using a Pearson Correlation Test (r = 0.98, p < 0.01). A linear relationship between change in fluorescent cells and changes in confluence was observed until approximately 250% increase in confluence [file 12935_2020_1625_MOESM1_ESM.tiff]

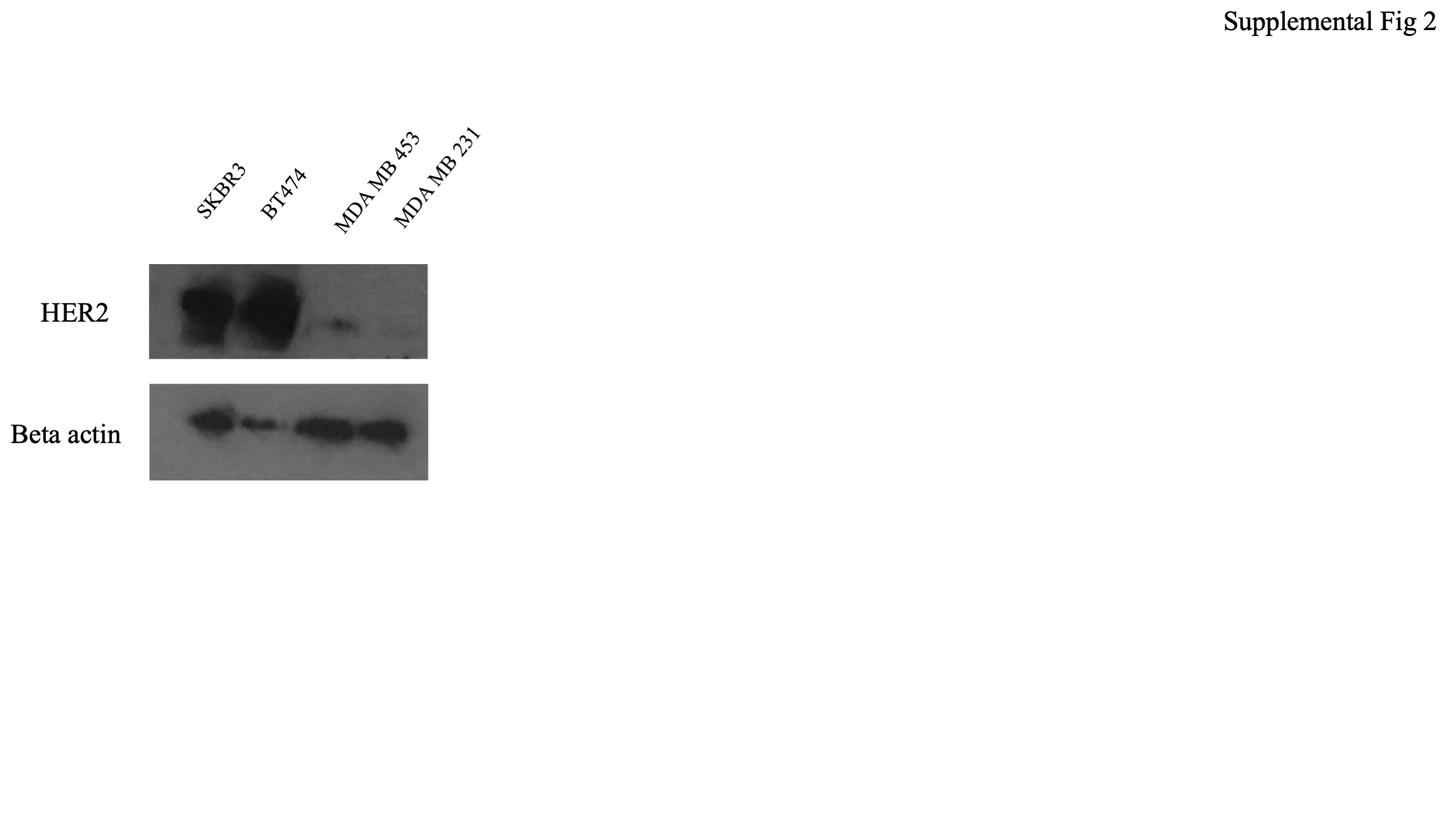

Supplement: Supplementary file 2 — Additional file 2: Figure S2. Western blot of HER2 expression in breast cancer cell lines. Western blot of HER2 expression in breast cancer cell lines. Breast cancer cell lines were probed for HER2 expression through western blot. High HER2 expression was observed in BT474 and SKBR3 cell lines. Moderate HER2 expression was observed in MDA-MB-453 cell line. Low HER2 expression was observed in MDA-MB-231 cell line [file 12935_2020_1625_MOESM2_ESM.tiff]
